# Grid-texture mechanisms in human vision: Contrast detection of regular sparse micro-patterns requires specialist templates

**DOI:** 10.1038/srep29764

**Published:** 2016-07-27

**Authors:** Daniel H. Baker, Tim S. Meese

**Affiliations:** 1 Department of Psychology, University of York, York, YO10 5DD, UK; 2 School of Life & Health Sciences, Aston University, Birmingham, B47ET, UK

## Abstract

Previous work has shown that human vision performs spatial integration of luminance contrast energy, where signals are squared and summed (with internal noise) over area at detection threshold. We tested that model here in an experiment using arrays of micro-pattern textures that varied in overall stimulus area and sparseness of their target elements, where the contrast of each element was normalised for sensitivity across the visual field. We found a power-law improvement in performance with stimulus area, and a decrease in sensitivity with sparseness. While the contrast integrator model performed well when target elements constituted 50–100% of the target area (replicating previous results), observers outperformed the model when texture elements were sparser than this. This result required the inclusion of further templates in our model, selective for grids of various regular texture densities. By assuming a MAX operation across these noisy mechanisms the model also accounted for the increase in the slope of the psychometric function that occurred as texture density decreased. Thus, for the first time, mechanisms that are selective for texture density have been revealed at contrast detection threshold. We suggest that these mechanisms have a role to play in the perception of visual textures.

Much has been written about the improvement of contrast sensitivity with stimulus area for periodic grating stimuli^1^,^2^,^3^,^4^. Recent work^3^,^4^,^5^,^6^ estimates that higher order mechanisms pool the activity of typical V1 cortical cells (with receptive fields in the order of 1 to 2 cycles) across as many as 12 grating cycles^7^,^8^, or more^9^. But such studies have typically been conducted with spatially contiguous stimuli. While real world examples of such stimuli can be found (e.g. picket fences), natural images are rich in textures (e.g. the leaves on a tree) that are much more complex in character (e.g. the texture density of leaves depends on the time of year). At present, very little is known about the mechanisms and strategies involved in the contrast detection of such sparse textures. Under some models and situations, summing uniformly across such stimuli is a questionable strategy because for low-density stimuli, the impact of internal noise can offset the benefit of signal summation. Knowing more about this is important, since one might suppose that the perception of texture density^10^/numerosity^11^—key mid-level processes in the perceptual hierarchy—will involve related processes and/or mechanisms to those involved in the detection task.

A recent series of experiments has measured thresholds for stimuli with regularly spaced gaps that remove half of the contrast area^3^,^5^,^6^,^7^,^9^,^12^. (By ‘contrast area’, we mean the integral of luminance contrast over the entire stimulus region.) Summation ratios were calculated by comparing sensitivity between these and contiguous stimuli. They were found to be up to a factor of 2 (i.e. the gaps halved contrast sensitivity as well as contrast area), being largest for the smallest gaps^6^. A summation ratio of 2 implies a linear summing process that is free of influences from contrast nonlinearities and the integration of internal noise. Detailed modelling attributed this to signal combination within individual filter-elements (e.g. within V1 receptive fields) for the small gaps^6^. For larger gaps, longer range summation across filter-elements (of common spatial frequency and orientation) was also evident, producing a quadratic effect, attributable to a squaring nonlinearity at the output stage of each filter-element prior to the long-range summing stage^6^ (see also^4^). Importantly, model observers pooled over both the signal and gap regions in the stimuli in the studies above, such that the total level of internal noise depended on stimulus diameter (or width), not contrast area. This mandatory pooling assumption is supported by the finding that it is not possible to identify the presence of gaps at contrasts around detection threshold^3^.

In the present study we investigated the process of area summation of contrast signals at detection threshold for regular texture stimuli that varied in the sparseness of their micro-patterns (we call these ‘grid-textures’). We systematically varied the ratio of marks (small stimulus elements) to spaces (or ‘gaps’, see Fig. 1a for examples), as well as the area (i.e. overall size) of our grid-textures. Thus, to try and optimize performance, our observers were faced with the problem of combining the evidence for signal across space. The problem is not trivial because one must also consider the deleterious effects of internal noise. At one extreme (often taken to be a minimum combination rule^13^, akin to what is sometimes called probability summation^14^) the observer might simply monitor their noisy neural activities across their spatial representation, picking the largest (MAX) response as the best evidence for signal in that stimulus interval. This has the advantage that irrelevant noise is not accumulated over space into the decision variable, but the disadvantage that there is little benefit from increasing the number of local signals in the display (e.g. from increasing overall stimulus size). Another possibility is to sum over the entire stimulus region. This has the benefit that weak signal information is accumulated, but the downside that early internal noise is also accumulated. In fact, for contiguous stimuli of known size, this is the ideal thing to do^13^, but this strategy might be disadvantageous for our grid-textures, some of which contain few signals spread over space, but a great deal of internal noise because of the large gaps. A third possibility is that the observer might try to match a template of the grid-texture to the stimulus; this is a sophisticated strategy, and is ideal in the sense that it maximises the potential for signal integration without also integrating internal noise beyond that which accompanies the local signals. Thus, our experiment presents a strategic challenge to our observers with the possibility of revealing previously unseen details of the underlying visual architecture used in contrast detection.

However, there is a major technical difficulty in conducting experiments using spatially extensive stimuli. There is a reduction in contrast sensitivity with eccentricity that is substantial^15^,^16^, meaning that for stimuli with diameters beyond around 12 grating cycles, adding extra cycles barely improves performance^4^. Thus, for very sparse textures (e.g. the rightmost panel in Fig. 1a) very little of the stimulus energy will be detectable.

To ameliorate this problem we normalized the contrast of our stimulus elements using detailed measurements of binocular sensitivity across the visual field^9^,^16^ for the same three observers who took part in the study here. (The form of the sensitivity function across the visual field is known as a witch’s hat^16^, owing to its shape when shown as a surface plot.) These contrast-compensated stimuli (e.g. Fig. 1c) were designed to equate sensitivity to the individual micro-elements across the entire display, providing us the best chance of characterising the spatially long-range signal detection processes for our texture patterns. To interpret our results, we compared them to the predictions of several computational models.

## Methods

### Apparatus and Stimuli

Stimuli were displayed on a Nokia MultiGraph 445X monitor using a ViSaGe stimulus generator (Cambridge Research Systems Ltd., Kent, UK) running in 14-bit mode. The monitor was gamma corrected using a photometer, and had a mean luminance of 60cd/m^2^ and a refresh rate of 120Hz.

**Figure 1:**
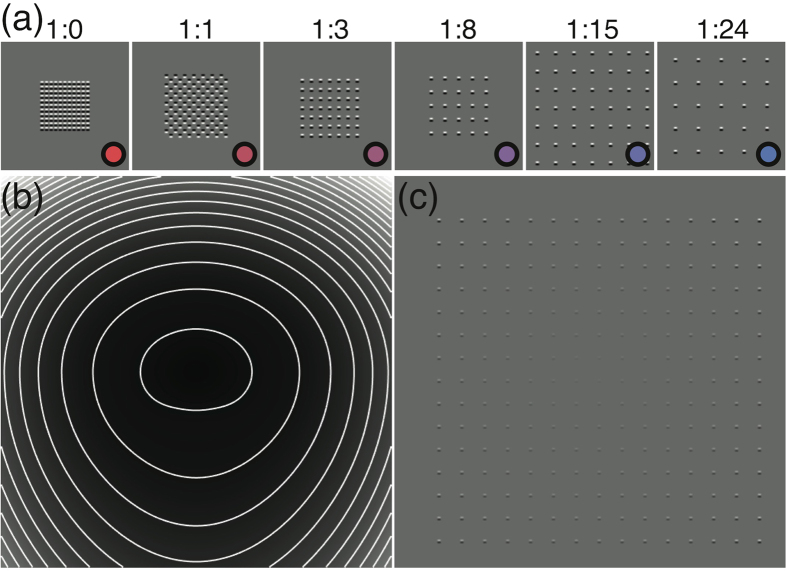
(**a**) Example grid-textures with various mark:space ratios. The coloured circles correspond to the symbols used in later figures. Note that the stimulus area (overall size) is smallest for the stimulus on the left, and larger for the two stimuli on the right. (**b**) An example of an inverse attenuation surface (also known as an inverse ‘witch’s hat’) by which the stimulus contrasts were multiplied to compensate for the decline in sensitivity with eccentricity (these were derived independently from Baldwin *et al*.^16^ for each observer). White lines indicate contours of constant attenuation, and were not present in the surface by which the stimuli were multiplied. (**c**) An example of a contrast-compensated grid-texture. Viewed at the correct distance on a calibrated display, each element is displayed at the same multiple of its detection threshold.

Stimulus elements were single cycles of a horizontal sine-wave grating with a spatial frequency of 4c/deg. The elements were curtailed in the horizontal direction by a half-wave rectified sine-wave of half the carrier frequency^6^. The square grid-textures were of various sizes, and various densities (always with regular spacing between elements), which we report as mark:space ratios. High contrast examples are shown in Fig. 1a.

To compensate for the decline in sensitivity across the visual field we adjusted the local contrasts of our stimuli as a function of eccentricity. This normalization was based on measurements from a previous study^16^ that were used to produce an inverse ‘attenuation surface’ (see Fig. 1b for an example) by which the stimuli were multiplied. This normalization procedure was performed independently for each observer. A high contrast example of the result of this normalization is shown in Fig. 1c. This general approach has been validated in a separate study using grating stimuli^9^.

### Procedure

Observers sat 60 cm from the display with their head in a chin rest and viewed the display binocularly. The experiment was blocked by mark:space ratio and stimulus area, and involved a total of 36 conditions (6 ratios by 6 areas). The areas for each mark:space ratio were chosen to keep the stimuli in a regular square configuration. The spatial extent of each stimulus region was indicated by a quad of small surrounding points that also served to aid fixation. For mark:space ratios greater than 1:0 the spatial position of the entire grid stimulus was randomly jittered from trial to trial (in integer multiples of element size in the x- and y-directions) such that, on average, target elements were evenly distributed across the display region. This meant that extrinsic spatial uncertainty increased in direct proportion to the mark:space ratio (i.e. the number of different spatial positions of the grid increased with mark:space ratio).

Stimuli were presented for 100 ms in a two-interval forced-choice (2IFC) experiment. In one interval, selected at random, the stimulus contrast was determined by a pair of independent 3-down-1-up staircases with a step size of 3dB (a factor of 1.41). In the other interval, the stimulus contrast was 0. Both intervals were indicated by auditory beeps, and the inter-stimulus interval was 400 ms. Observers used a mouse to indicate which of the two intervals they believed contained the stimulus, and the pitch of a further beep informed them if they were correct. After a preliminary homing in stage (for which the data were discarded), each staircase terminated after 12 reversals or 70 trials, which ever came first. Experimental data were then combined across the pair of staircases, producing empirical psychometric functions from 92 trials, on average.

**Figure 2:**
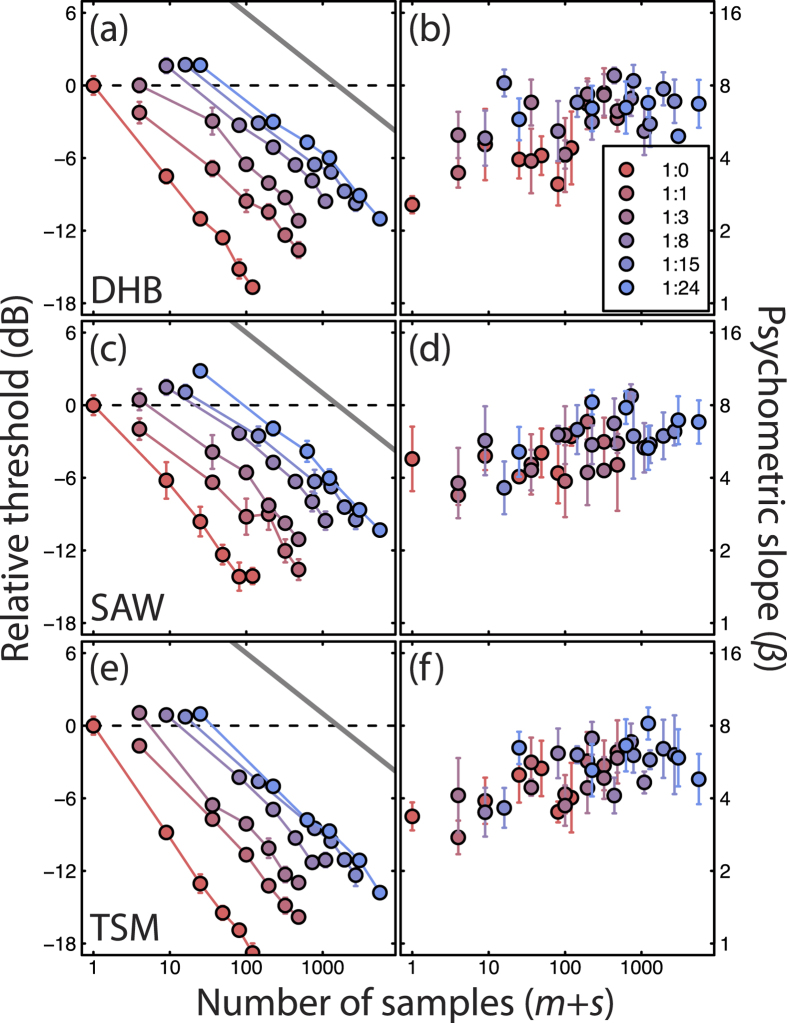
Contrast detection thresholds (**a,c,e**) and psychometric slopes (**b,d,f**) for grid-texture stimuli for three observers (different rows). All results are plotted as a function of the number of samples (*m*+*s*, where *m* is the number of target elements and *s* is the number of space elements, or gaps, and is proportional to the total area bounded by the quad of fixation marks), with different colours representing different mark:space ratios (legend). The threshold data were normalized to the leftmost point for each observer (dashed lines). Error bars show ±1SE across repetition of the experiment, and in many cases are smaller than symbol size. The diagonal grey lines have a slope of −1/4 and are included as a visual comparison against the empirical summation slopes. The total number of psychophysical 2IFC trials is approximately 34,500.

**Figure 3:**
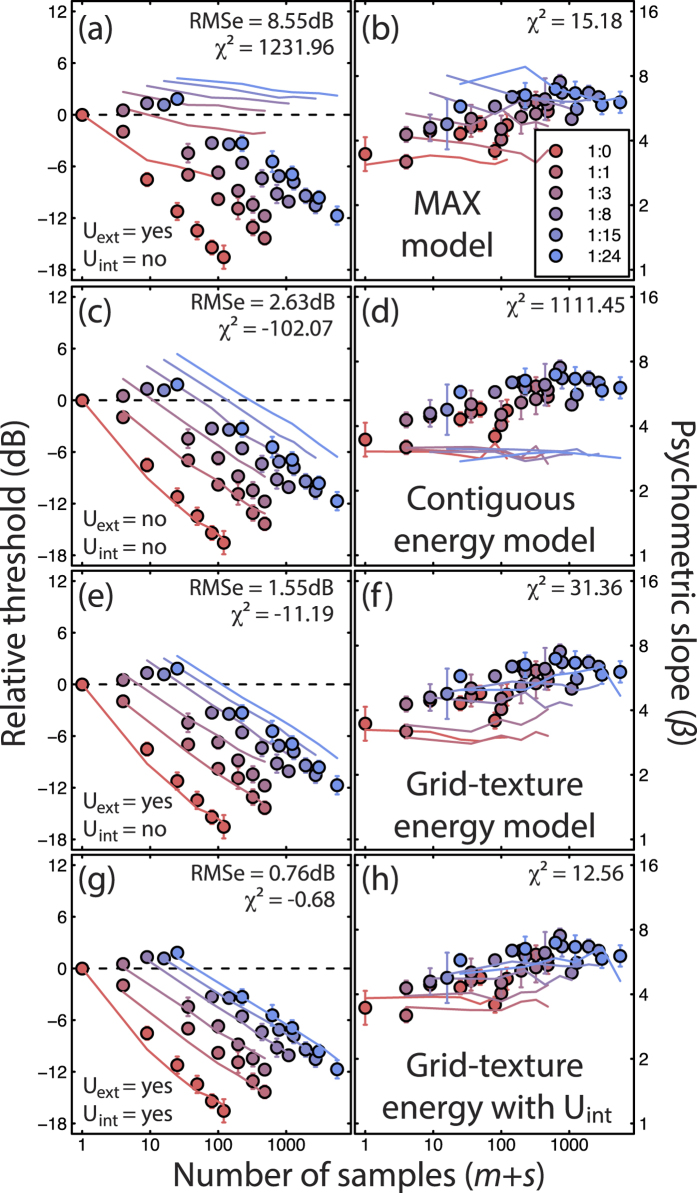
Various model predictions (different rows) and human results (repeated across each row) averaged across three observers (these are the same human results as those in Fig. 2). Left column: contrast detection thresholds; right column: the slopes of the psychometric functions. The curves are model predictions based on stochastic simulations with 1000 trials per target contrast level. Error bars represent ±1SE across observers. RMS and χ^2^ error statistics indicate the goodness of fit within each panel. The terms U_ext_ and U_int_ in each row indicate whether extrinsic and fixed intrinsic uncertainty were properties of each model. Notice how extrinsic uncertainty is needed to capture the psychometric slope effects, and that by adding a fixed level of intrinsic uncertainty, the effects of extrinsic uncertainty are reduced (the model curves are less spread out in (**h**) than in (**b**) and (**f**). Note also that the non-monotonicity of some of the model functions (particularly the psychometric slopes) derives from their stochastic implementation.

Each condition was repeated four times, and the results from each block were fitted with a cumulative log-Gaussian psychometric function by probit analysis to estimate the threshold (75% correct) and slope (equivalent Weibull *β*, calculated as *β* = 10.3*/σ*, where *σ* is the standard deviation of the Gaussian). Thresholds and slopes were averaged across the four repetitions to produce the plots in Fig. 2, and across three observers to produce the average results used for modelling (Fig. 3). Thresholds are expressed in decibels (dB), defined as *C*_*dB*_ = *20log*_*10*_(*C*_*%*_), where *C*_*%*_ is Michelson contrast in percent, defined as *C*_*%*_ = (*L*_*max*_ − *L*_*min*_)/(*L*_*max*_ + *L*_*min*_) where *L* is luminance. Raw experimental data are available at: http://dx.doi.org/10.6084/m9.figshare.1587105.

### Observers

Three observers completed the experiment. Two were authors, and all were psychophysically experienced. Observers wore their normal optical correction if required during testing. Experimental protocols (050507/NL5) were approved by the Life and Health Sciences Ethics Committee at Aston University, and the experiments here were carried out in accordance with these. Participants gave written informed consent before testing began.

### Modelling

To reveal the basic visual mechanisms underlying the detection of our stimuli, we tested several computational models, refining them in light of their predictions compared with the human results. In the main, we were interested in comparing what is sometimes called *signal selection* (or probability summation, implemented here by a MAX operator^6^,^14^,^17^) with *signal combination*, involving the linear summation of spatial signals over area^4^,^6^, under various assumptions about integrator templates and intrinsic uncertainty. We also compared models having a linear transducer with those having a square-law transducer. (We have investigated the exponent of the contrast transducer before (*p* in *r*^*p*^, where *r* is the local contrast response), and concluded that it is equal, or very close, to, *p* = 2 (i.e. a square-law, consistent with energy detection)^4^,^5^,^6^,^9^. Thus, there was little reason to allow the exponent to be a free parameter in the modelling here, the comparison to a linear transducer serving mainly as a reality check. Based on our informal observations and previous analyses we think it very unlikely that our conclusions would be substantially different had this parameter been free). All models were implemented in Matlab for images at the resolution used in the experiments (24 pixels per degree of visual angle). We modelled the detection process stochastically, and simulated 1000 trials for each stimulus contrast level. On each trial, we computed a pair of numbers to represent the activity in the two trial intervals. The larger of the two numbers determined the interval chosen by the model observer as the one most likely to contain the target. Because the models did not suffer a loss of contrast sensitivity with eccentricity, there was no need to apply the contrast compensation described above to the model stimuli. All models featured filtering in the Fourier domain using Cartesian separable log-Gabor filters^6^ with bandwidths of ±25° and 1.6 octaves. Filter orientation was matched to the orientation of the micro-patterns, which was always horizontal.

To avoid any potential confusion, we wish to point out (as described above) that in all of our models, the model observer computes the maximum of some decision variable across the two temporal 2IFC intervals. In our terminology here (and elsewhere) this is not what we mean by a MAX model. A MAX model is one in which the observer is picking the largest response across a set of noisy mechanisms *within* a stimulus interval. This process is sometimes referred to as *probability summation*, but we are reluctant to use that term here since, strictly speaking, that term is tied to (the now discredited^9^,^18^) high threshold theory, where the probability of a detector being in a ‘detect state’ is summed over mechanisms according to the conventional rules of probability statistics. That theoretical position is not consistent with the signal detection approach used here, for which there are no ‘states’ of detection, but local responses that depend on external signal, transduction and internal noise.

The stochastic model simulations were computationally intensive, so parameter optimization was not feasible (i.e. when single free parameters were explored, this was done by hand, as outlined in the model predictions section of Results). To remove differences in absolute sensitivity between model and humans, we normalized both the threshold data and model predictions to the single element condition for a mark:space ratio of 1:0 (e.g. the leftmost point in Fig. 3a,c,e,g). We assessed goodness of fit for thresholds by calculating the RMS error (root mean square error) in dB between each model and the average threshold data. We also calculated χ^2^ statistics both for threshold and slope predictions. Because distributions of psychometric slopes (*β*) are approximately logarithmic^19^ we log-transformed the slope values first.

### Uncertainty

An important component in signal detection is observer uncertainty^20^. On the assumption that all of the visual mechanisms that contribute to the observer’s decision variable are noisy, then uncertainty will increase the impact of internal noise by introducing irrelevant mechanisms into the detection process. The size of this effect increases approximately logarithmically with the level of uncertainty^20^. For the experiments and modelling here there are two forms of uncertainty to consider. Extrinsic uncertainty relates to uncertainty about the details of the stimulus that is to be detected (e.g. its position on the display) and, therefore, which visual mechanisms should be monitored. Intrinsic uncertainty relates to non-ideal behaviour in which the observer needlessly recruits irrelevant visual mechanisms in constructing the decision variable. (For further details see^4^,^17^,^20^). We also consider two functional forms of intrinsic uncertainty. In *fixed* intrinsic uncertainty, the number of irrelevant mechanisms (a free parameter) is fixed across all stimulus conditions. In *proportional* intrinsic uncertainty^4^, the number of irrelevant mechanisms (a free parameter) is proportional to the area (signal + gaps) of the stimulus. Note that in all cases, the irrelevant mechanisms in the models have the same gains and noise characteristics as the potentially relevant ones. In general, an increase in uncertainty (regardless of whether it is intrinsic or extrinsic) will decrease sensitivity and increases the slope of the psychometric function.

## Results

Results for all three observers are shown in Fig. 2, with contrast detection thresholds and psychometric slopes shown in the left and right columns, respectively. We plot the results as a function of the number of discrete ‘samples’ (each the size of one element) within the stimulus area. This is the number of target elements (marks, *m*), plus the number of gaps (spaces, *s*) between them (i.e. *m* + *s*). For the 1:0 ratio, where there were no spaces (*s* = 0), this is equivalent to a classical area summation function. Note also that the total number of samples (*m* + *s*) for the smallest stimulus (top left points of each threshold function) increases with mark:space ratio because the number of gaps (spaces, *s*) also increases with mark:space ratio, even though the number of target elements is fixed (*m* = 1). The exception to this is the 1:1 ratio for which there were two target elements (*m* = 2) to maintain the square formation.

For the mark:space ratio of 1:0 the results replicate the classical effect of increasing the stimulus area of a grating^1^,^4^, where thresholds improve as a function of area. However, because the stimulus contrast was adjusted to compensate for the reduction of sensitivity with eccentricity (see Methods), these area summation functions do not asymptote at larger sizes as is typically reported for uniform contrast stimuli^4^, but are fairly straight lines on double-log co-ordinates, broadly consistent with a power-law relation between area and sensitivity, implying that our contrast compensation method (Fig. 1c) was effective. The solid grey curves in Fig. 2 have a slope of −1/4 on these double log axes (i.e. they are given by a fourth-root power-law exponent of −0.25) consistent with the overall summation slopes in our results here, and also previous results where the compensation method has been used with gratings^9^. Broadly speaking, this fourth-root rule is consistent with each of the following interpretations: (i) probability summation under high threshold theory where the slope of the psychometric function is *β* = 4, (ii) a contrast transducer exponent of *p* = 4 prior to spatial summation and additive limiting noise and (iii) a cascade of quadratic effects from a square-law (*p* = 2) transducer and the linear summation of signal and noise over space. Detailed analysis and discussion by Baldwin and Meese^9^ clearly favours the third interpretation over the others and has served further as a rejection of high threshold theory under the assumptions of probability summation. The cascade of quadratic effects in that preferred interpretation is also a property of the successful model developed here.

The threshold functions are approximately parallel across the range of mark:space ratios, though the sparse stimuli (ratios lower than 1:0) appear to be slightly shallower than the 1:0 function (left most curves), over the initial part of the function at least. In our models (see below), this is due to the effects of summation within individual filter-elements for the denser textures^4^,^6^; it seems likely that the behavioural effect is similarly attributable to spatial blurring by neural receptive fields with footprints larger than a single stimulus element.

The slight increase in threshold as a function of mark:space ratio for the first point in each function (top-left point in each function) shows the effect of spatial uncertainty, as we now explain. Each of these points represents the threshold for a single element (apart from the 1:1 ratio for which *s* = 2 (see above), explaining why sensitivity for that condition falls out of sequence with the others). The target for the 1:0 ratio was always placed at the same location, so there was no extrinsic uncertainty, whereas in the other conditions, there were several potential spatial locations owing to the spatial jitter (see Methods) and so the observer was extrinsically uncertain. Consistent with previous reports^4^, the effect of small levels of uncertainty at threshold were weak but measurable—thresholds for the single element condition increased slightly at higher mark:space ratios—, suggesting that intrinsic uncertainty was also very low, otherwise it would have swamped the extrinsic effects (see^4^ for details; see also^5^).

The slope of the psychometric function for a single element of known position (i.e. the leftmost point) was around *β* = 3 to 4, consistent with typical reports^19^. Slopes became gradually steeper across the plot, reaching values of around *β* = 8 for the largest mark:space ratios. Although the variance was too large to determine whether this increase was due to overall stimulus area or the mark:space ratio, previous studies have ruled out an increase in psychometric slope with area^19^,^21^. We therefore attribute this effect to the mark:space ratio, which is also predicted by some of the models in the following section.

### Model predictions

We present four model arrangements, only one of which provided good accounts of both the threshold and slope results. Each of the three unsuccessful models could be modified in several ways to try and improve their performance; we tried various combinations of different transducer exponents (*p*), different decision rules, different levels and forms of intrinsic uncertainty and so on. (Note that in general, the slope of the psychometric function is known to increase with each of the following: (i) the exponent of the contrast transducer and (ii) the level of uncertainty.) As none of these modifications was able to produce either qualitatively or quantitatively acceptable predictions, we present only the basic versions of the unsuccessful models, thereby allowing us to illustrate the development of the key components in the successful model.

The first model (the MAX model, sometimes called a probability summation model^14^) takes the maximum local response across the filtered stimulus, after nonlinear transduction if present (in this case, squaring) and the addition of zero-mean Gaussian noise to each pixel of the filtered image. The model observer monitors only the display region that contained the stimulus (e.g. that which was indicated by the fixation quad during the experiment). However, since this means that the observer uses irrelevant locations in the sparse stimulus conditions, this model observer is inherently extrinsically uncertain. Predictions (curves) are shown in Fig. 3 for thresholds (Fig. 3a) and psychometric slopes (Fig. 3b), compared with the experimental results (from Fig. 2) averaged across all three observers (circles). The fit of the model for thresholds is very poor, with an RMS error exceeding 8dB. In general, the MAX model predicts too little summation in all conditions. On the other hand, the predictions for the slope of the psychometric function are qualitatively very good, showing a slight increase in *β* with mark:space ratio owing to a concomitant increase in extrinsic uncertainty.

Because the predictions for the psychometric slopes were good, we tried to improve the threshold performance in various ways. Replacing the square-law transduction with a linear transducer increased the levels of summation, making the summation slopes steeper, but not steep enough, and caused the overall fit to degrade (RMS error of 9.52dB) because the decline in sensitivity at high mark:space ratios became far too severe (not shown). Also, the linear transducer made the psychometric slopes far too shallow (*β* < 2; see^4^). We then tried adding a high (fixed) level of uncertainty to all the conditions, since this is known to steepen the lower part of the summation slope^4^. This also had the benefit of steepening the psychometric slopes back to appropriate levels, but it had the unfortunate side effect of causing each of those functions to become less steep as area increased, contrary to our results. We tried, but could find no modifications that allowed a variant of the MAX model to provide a convincing account of the human data. Following previous failures of the MAX model in more conventional area summation experiments^4^ this is perhaps not surprising; presumably, as for gratings, our texture detection task involves some form of signal combination.

We then considered a model that summed contrast energy over space. In previous work we have called this the combination model^6^ or the noisy energy model^4^, as it sums the squares of the local filtered signals as well as the local noise sources (zero-mean Gaussian internal noise). Since it does this over a uniform template that is matched to the overall stimulus area, we refer to this model here as the *contiguous energy model*. This integration strategy means that internal noise grows with stimulus area, but because the locations of the target elements within the stimulus area are irrelevant, there is no extrinsic uncertainty. This model has been very successful in accounting for contrast detection performance in several recent studies^3^,^4^,^5^,^6^,^9^.

The contiguous energy model did a much better job in describing the thresholds (see Fig. 3c) than did the MAX model, producing an RMS error of 2.63dB and improving the χ^2^ statistic by an order of magnitude. In particular, the predictions were very good for the 1:0 and 1:1 mark:space ratios (red symbols and curves) which correspond to conditions explained previously by this model^6^. However, there were clear inconsistencies. For one, the model predicts much higher thresholds for the larger mark:space ratios than we measured empirically. This was surprising, since the real observers were outperforming the otherwise successful signal integration model. Furthermore, the predictions for the psychometric slopes (Fig. 3d) failed to capture the increase in slope with mark:space ratio seen in the human data, producing the worst χ^2^ value for the psychometric slopes in any of the four models.

We attempted several modifications to this model (not shown) to improve its performance, including adding various amounts of (fixed) intrinsic uncertainty^4^. This had the effect of increasing thresholds for low mark:space ratios relative to those for higher ratios, improving the quantitative fit somewhat. However, it made little difference to the slope predictions, which remained invariant across conditions (see Fig. 2d). We also tried a form of intrinsic uncertainty in which a fixed number of uninformative channels that matched the target area were monitored by the model observer. This produces proportional intrinsic uncertainty since the number of irrelevant mechanisms increases with stimulus area. This had no effect on relative thresholds (though absolute thresholds increased with uncertainty), but produced a vertical shift in the predicted psychometric slopes. A model such as this might be developed to improve the quantitative fit (by optimising the level of uncertainty), but would fail to account for the empirical increases in psychometric slopes with mark:space ratio.

How then can we account for both the threshold and slope results in a single model? Since the MAX model gives a good account of the psychometric slopes, and the contiguous energy model gives a fair account of thresholds and the spatial summation effects, we might expect a successful model to be derived from a combination of the relevant features of the two. We constructed a model that summed contrast energy (the square of local contrasts plus noise) only within a template of the stimulus (i.e. within templates that ignored the gaps in the stimulus structure, and hence the noise from those locations), and took the MAX response across a family of such noisy grid-templates covering all of the possible spatial locations of the stimuli (i.e. we explicitly accommodated the trial-to-trial spatial jitter/extrinsic uncertainty in the experiment). For expedience, we used model templates that were the filtered and squared stimulus images for each spatial location, though overall, their precise form is much less important than the footprint of their texture element positions.

The *grid-texture energy model*, above, does a good job of describing both the thresholds and psychometric slopes (Fig. 3e,f), producing an RMS error of 1.55dB for the thresholds. It slightly overestimates thresholds for large mark:space ratios, and slightly underestimates slopes across most conditions. To try and rectify this we added small amounts of fixed intrinsic uncertainty (Fig. 3g,h). This might, for example, represent inappropriate attention to other mechanisms not tuned to the orientation and spatial frequency of our targets^13^, or perhaps distraction^22^, by factors extraneous to the experiment. We found that including just three irrelevant mechanisms in all conditions fixed both shortcomings of the ‘certain’ version of the grid-texture energy model. This is strikingly consistent with previous estimates of intrinsic uncertainty from experiments using grating-type stimuli^5^. This version of the model (Fig. 3g,h) produced the smallest error statistics for both thresholds and slopes. In particular the threshold fit was excellent, with an RMS error of 0.76dB. The level of intrinsic uncertainty was the only free parameter in this model, and was optimised by hand. Thus, one overall conclusion from the experiments here and elsewhere (in which we have fitted intrinsic uncertainty and the contrast transducer, constrained by manipulating extrinsic uncertainty and spatial parameters of the stimulus^4^,^5^,^6^) is that the contrast transducer is a square-law, consistent with energy detection, and that intrinsic uncertainty is modest.

## Discussion

We measured contrast detection thresholds for uniform periodic two-dimensional texture stimuli (grid-textures) with various levels of sparseness. When sparseness was high, human performance was better than predicted by models that either maxed (sometimes called probability summation) or summed over the signal and internal noise, including the inter-element internal noise. A much more successful model excluded noise between elements by using templates matched to the regular grid-texture of the stimulus. We discuss the implications of this model for our understanding of area summation of luminance contrast, and the perception of texture density/numerosity.

### Area summation of luminance contrast

The stimuli with the two smallest mark:space ratios (1:0 and 1:1) correspond directly to the *j* = 0 and *j* = 1 ‘Battenberg’ stimuli of Meese^6^. Consistent with that study, we found a vertical offset of around 6dB (a factor of 2) between the 1:0 and 1:1 area summation functions (compare the vertical difference between the first two functions in each of Fig. 2a,c,e), particularly for the larger stimulus areas. This substantial offset is produced by all of the models in Fig. 3. On the face of it, this is perhaps surprising; for example, the summation model pools in noise from the gaps in the 1:1 stimuli, whereas the templates should ignore these uninformative regions. However, in practise, the spatial filtering spreads the stimulus energy across space to such an extent that the template (which incorporates the filtering effects) is very similar for both 1:0 and 1:1 stimuli, which means that the sum of the internal noise is also very similar across these two sparseness conditions. Another possibility is that texture templates are simply not used in the 1:1 condition (perhaps they don’t exist for that density), leaving contiguous integration as the best available strategy. Either way, we attribute the strong (~6dB) of summation seen across these conditions to linear summation within V1-type spatial filter-elements, as we have done before^6^.

Previous work^4^,^6^,^7^ has concluded that luminance contrast energy is summed over contiguous regions of the visual field for contrast detection of sine-wave gratings and mark:space ratios of 1:0 and 1:1. That conclusion is not challenged here, but we have now shown that a more effective strategy is available to the observer at lower mark:space ratios where the contiguous spatial integration strategy becomes increasingly inefficient. The fact that our observers outperformed the earlier models that we considered implied that we needed model templates more closely tailored to our sparse grid-textures in order to reject contributions from irrelevant local noise sources. When the known effects of extrinsic^4^ and intrinsic^5^ uncertainty were also included, this grid-texture energy model captured all aspects of our results.

### Mechanisms for representing texture regularity

Morgan and MacLeod^23^ have recently extended earlier luminance texture work, which used suprathreshold white dots^24^, to suprathreshold modulation of luminance-balanced dot displays. They found that suprathreshold modulations of contrast and density/numerosity summed to detection threshold, suggesting a visual mechanism for the modulation of contrast energy that is indifferent to the source of modulation (dot contrast or dot density). This provides further evidence for the importance of contrast energy in texture perception. Morgan *et al*.^25^ presented observers with square arrays of Gaussian blobs, and jittered the positions of the individual elements. They found that with small amounts of jitter, observers perceived the array as being regular, even when it was not. They concluded that the brain represents a regular template for a pattern, rather than the veridical pattern itself. This template masks small differences in positional jitter (i.e. internal or external position noise), presumably as a way of reducing the dimensionality or complexity of the stimulus. Furthermore, the perceived regularity of such textures is prone to adaptation^26^, a finding that also implies dedicated mechanisms. The texture template model that we propose here is entirely consistent with these results. Whether there is a fixed number of such templates, or whether they are constructed on-the-fly to meet the demands of a particular task, remains unresolved. However, the finding that our model requires probability summation (maxing) across multiple mechanisms implies that if they are constructed on-the-fly, then many such mechanisms are constructed simultaneously.

Our experiments here do not contribute to the debate on the distinction between numerosity and texture perception, though we note that Morgan *et al*.^27^ have proposed an energy model as the basis for performing relative numerosity judgements. It seems likely that our texture templates, with various diameters and centre-to-centre distances between elements, are also good candidate mechanisms for numerosity/density judgements, at least for regular patterns. Of course, the response of an individual mechanism would be classically ambiguous, but a population code across a set of mechanisms selective for different sizes, densities, and possibly other dimensions not considered here, might well be useful. We note, however, that very different types of mechanisms have also been proposed for this type of task. For example, Dakin *et al*.^28^ propose that density is encoded by the relative responses of high and low spatial frequency filters, which are responsible for picking up number and size, respectively.

At the outset of the work here we had not anticipated revealing the exotic visual mechanisms that we have reported using a paradigm as prosaic as contrast detection. Indeed, within spatial vision, and with the exception of a few forays into the investigation of elongated (contour type) mechanisms^29^,^30^, contrast detection thresholds have been used to characterise either (i) entire system properties^31^ or (ii) the basic mechanisms of early vision, usually attributed to the small receptive fields in V1^32^ or a combination of the two^33^. The witch’s hat^16^ (the attenuation surface) was key to the experiments here, of course, since only by normalising sensitivity across the visual field is it possible to reveal the properties of spatially extensive mechanisms at contrast detection threshold, should they be there to be found. We are left wondering then, what other mid-level visual mechanisms might be revealed at threshold now that the benefits of the witch’s hat have been demonstrated.

## Additional Information

**How to cite this article**: Baker, D. H. and Meese, T. S. Grid-texture mechanisms in human vision: Contrast detection of regular sparse micro-patterns requires specialist templates. *Sci. Rep.* **6**, 29764; doi: 10.1038/srep29764 (2016).
